# DecoyFinder, a tool for finding decoy molecules

**DOI:** 10.1186/1758-2946-4-S1-P2

**Published:** 2012-05-01

**Authors:** Cereto Massagué Adrià, S Garcia-Vallvé, G Pujadas

**Affiliations:** 1 Tarragona, Spain

## 

DecoyFinder is a graphical tool which helps finding sets of decoy molecules for a given group of active ligands. It does so by finding molecules which have a similar number of rotational bonds, hydrogen bond acceptors, hydrogen bond donors, logP value and molecular weight, but are chemically different, which is defined by a maximum Tanimoto value threshold between active ligand and decoy molecule MACCS fingerprints. Optionally, a maximum Tanimoto value threshold can be set between decoys in order assure chemical diversity in the decoy set.

During the talk, the algorithm used by DecoyFinder in order to look for decoys sets will be described in detail and some examples of its application will be described and discussed.

