# Correction: DecoyFinder, a tool for finding decoy molecules

**DOI:** 10.1186/s13321-024-00809-0

**Published:** 2024-02-28

**Authors:** Adrià Cereto-Massagué, S. Garcia-Vallvé, G. Pujadas

**Affiliations:** Tarragona, Spain

**Correction: Journal of Cheminformatics 2012, 4(Suppl 1):P2** 10.1186/1758-2946-4-s1-p2

From 7th German Conference on Chemoinformatics: 25 CIC-Workshop Goslar, Germany. 6–8 November 2011

Following publication of the original article [1], we have been informed that first name and last name of the author Adrià Cereto Massagué has been switched.

The incorrect name is: Cereto Massagué Adrià

The correct name is: Adrià Cereto Massagué
